# Assessing Biofilm at the Bedside: Exploring Reliable Accessible Biofilm Detection Methods

**DOI:** 10.3390/diagnostics14192116

**Published:** 2024-09-24

**Authors:** Perry Mayer, Allie Clinton Smith, Jennifer Hurlow, Brian R. Morrow, Gregory A. Bohn, Philip G. Bowler

**Affiliations:** 1 The Mayer Institute (TMI), Hamilton, ON L8R 2R3, Canada; 2 Department of Honors Studies, Texas Tech University, Lubbock, TX 79409, USA; allie.c.smith@ttu.edu; 3 Consultant Wound Care Specialized Nurse Practitioner, Memphis, TN 38120, USA; jenny.hurlow@gmail.com; 4 College of Dentistry, University of Tennessee Health Science Center, Memphis, TN 38163, USA; 5 The American Professional Wound Care Association (APWCA), American Board of Wound Healing, Milwaukee, WI 53214, USA; 6 Independent Researcher, Warrington WA4 5QZ, UK; philbowler.consulting@gmail.com

**Keywords:** biofilm, chronic wounds, hard to heal wounds, bacterial fluorescence, biofilm blotting, wound infection

## Abstract

Introduction: Biofilm is linked through a variety of mechanisms to the pathogenesis of chronic wounds. However, accurate biofilm detection is challenging, demanding highly specialized and technically complex methods rendering it unapplicable for most clinical settings. This study evaluated promising methods of bedside biofilm localization, fluorescence imaging of wound bacterial loads, and biofilm blotting by comparing their performance against validation scanning electron microscopy (SEM). Methods: In this clinical trial, 40 chronic hard-to-heal wounds underwent the following assessments: (1) clinical signs of biofilm (CSB), (2) biofilm blotting, (3) fluorescence imaging for localizing bacterial loads, wound scraping taken for (4) SEM to confirm matrix encased bacteria (biofilm), and (5) PCR (Polymerase Chain Reaction) and NGS (Next Generation Sequencing) to determine absolute bacterial load and species present. We used a combination of SEM and PCR microbiology to calculate the diagnostic accuracy measures of the CSB, biofilm blotting assay, and fluorescence imaging. Results: Study data demonstrate that 62.5% of wounds were identified as biofilm-positive based on SEM and microbiological assessment. By employing this method to determine the gold truth, and thus calculate accuracy measures for all methods, fluorescence imaging demonstrated superior sensitivity (84%) and accuracy (63%) compared to CSB (sensitivity 44% and accuracy 43%) and biofilm blotting (sensitivity 24% and accuracy 40%). Biofilm blotting exhibited the highest specificity (64%), albeit with lower sensitivity and accuracy. Using SEM alone as the validation method slightly altered the results, but all trends held constant. Discussion: This trial provides the first comparative assessment of bedside methods for wound biofilm detection. We report the diagnostic accuracy measures of these more feasibly implementable methods versus laboratory-based SEM. Fluorescence imaging showed the greatest number of true positives (highest sensitivity), which is clinically relevant and provides assurance that no pathogenic bacteria will be missed. It effectively alerted regions of biofilm at the point-of-care with greater accuracy than standard clinical assessment (CSB) or biofilm blotting paper, providing actionable information that will likely translate into enhanced therapeutic approaches and better patient outcomes.

## 1. Introduction

Biofilms continue to be at the forefront of scientific research due to their profound implications across multiple fields, including medical, dental, and pharmaceutical spaces. They have been linked to many diseases in both human and animal hosts and pose a significant threat through the contamination of medical implants and equipment [1,2,3,4,5,6,7,8,9,10,11,12]. The actual magnitude of the impact of biofilms, both in human and economic costs, is likely underestimated due to under-detection. Nonetheless, recent estimates reveal a staggering annual global expenditure of approximately USD 5 trillion USD on biofilm management across various industries [13,14]. According to the US National Institute of Health, biofilms are implicated in over 80% of human microbial infections, presenting a significant threat as many of these infections remain unaddressed by standard antimicrobial treatments [1]. In chronic hard-to-heal wounds specifically, Malone et al. found that biofilms were present in up to 78% of cases [15]. Indeed, about a third of the USD 780 billion global expenditure on wound care in 2019 was linked to biofilm-related complications [15].

Biofilms are complex communities of microorganisms encased within a self-produced polymeric matrix. They are notoriously resilient to treatment due to their complex and adaptive nature. For example, the matrix offers a protected habitat and streamlines nutrient uptake and waste removal through a primitive circulatory system. Biofilms enable bacteria to develop unique traits otherwise absent in their free-floating planktonic state, including altered metabolism and gene expression, enhanced inter-cellular communication, and genetic transfer [16]. Persistent cells within biofilms, characterized by their transient dormancy and tolerance to antibiotics, contribute to treatment failure and recurrent infections [17,18].

In the case of hard-to-heal wounds, the precise role of biofilms remains a topic of debate. The “specific bacterial hypothesis” proposes that only certain species within polymicrobial biofilms are responsible for wound infection and delayed healing. In contrast, the “non-specific bacterial hypothesis” views the entire biofilm as collectively contributing to delayed healing [19,20]. However, conclusive evidence for either theory is lacking, suggesting that no single bacterial element or combination of elements consistently leads to a particular outcome in wound healing. From a clinical perspective, disrupting and removing biofilms appears to promote healing, independent of their etiology or pathogenic configuration [1,18,21,22]. However, there is a crucial need for sensitive methods to accurately identify and localize wound biofilm at the bedside, providing real-time feedback to guide its removal and/or disruption.

Most presently available modalities for biofilm detection rely on highly subjective clinical signs to signal more sophisticated laboratory-based methods. There is an urgent demand for reliable, straightforward, precise, and easily implementable diagnostic tools capable of detecting biofilms in wounds in real time. Enabling timely and effective biofilm eradication through an objective method at the bedside can shorten the time to heal and avoid complications, decreasing the human and economic burden of chronic wounds.

The present study aims to evaluate the diagnostic performance of bedside wound biofilm detection modalities against the combined results of standard scanning electron microscopy (SEM) and microbiology via quantitative Polymerase Chain reaction (PCR) and Next Generation Sequencing (NGS). Specifically, we assess three bedside techniques: assessment using clinical signs of biofilm (CSB), fluorescence imaging (MolecuLight^®^, Toronto, ON, Canada) for biofilm detection, and biofilm blotting (Saraya^®^, Osaka, Japan).

## 2. Material and Methods

### 2.1. Ethics and Registration

A prospective single-blind study (ClinicalTrials.gov No. NCT05196880) was conducted to evaluate the diagnostic accuracy of clinical signs of biofilm (CSB), bacterial fluorescence imaging (MolecuLight), and wound blotting (Saraya^®^, Osaka, Japan) against biofilm identification as validated by gold standard SEM imaging and microbiology. The study was approved by Veritas IRB (Montreal, QC, Canada) Ref # 2022-2907-9476-3. Participating subjects were informed of the study procedures and informed consent was obtained utilizing an IRB-approved form prior to any study-related procedures and data collection. Data were collected using a paper-based designated case report form and then transcribed into a password-protected centralized database (Microsoft^®^ Excel^®^ for Microsoft 365 MSO (Version 2408) for analysis. All data collection, processing, and analysis followed the principles of the code of ethics of the Declaration of Helsinki, as well as ICH GCP E6 R2.

### 2.2. Patient Population

Subjects were recruited between 24 February 2022 and 8 March 2023 from a specialized ambulatory wound care clinic (The Mayer Institute, Hamilton, ON, Canada). The included subjects were at least 18 years old, presenting with hard-to-heal diabetic foot, vascular, pressure, and neuropathic ulcers, and were willing and able to consent. Only one wound per patient was eligible for inclusion. Patients who did not fulfill the inclusion criteria or who were under an investigational drug treatment within a month of the enrollment date and/or had any contraindications for regular wound care (e.g., severely anti-coagulated and intolerable pain) were excluded.

**Figure 1:**
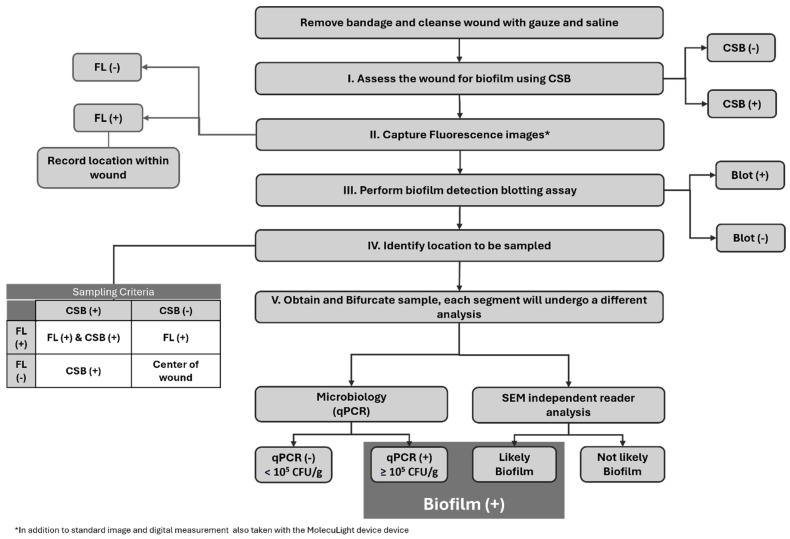
Overview of the study protocol. Flowchart describing the sequential order of the procedures performed on the study wounds. FL: Fluorescence Imaging, CSB: Clinical Signs of Biofilm, Blot: Biofilm Blotting, qPCR: quantitative Polymerase Chain Reaction, SEM: Scanning Electron Microscopy.

Overall, 45 subjects met the study criteria and were enrolled. During the study period, five subjects were excluded due to blood contamination of the specimen that rendered the SEM images ineligible. Consequently, the final analysis included 40 subjects for evaluation. Figure 1 sequentially describes the full study protocol. Given the prevalence of biofilm in hard-to-heal wounds, to increase the frequency of true negative biofilm samples for robust diagnostic accuracy calculations, we enrolled 20 study subjects considered to have clinical signs of biofilm (CSB) and 20 study-eligible subjects considered CSB.

### 2.3. Bedside Biofilm Diagnostic Methods

**Figure 2:**
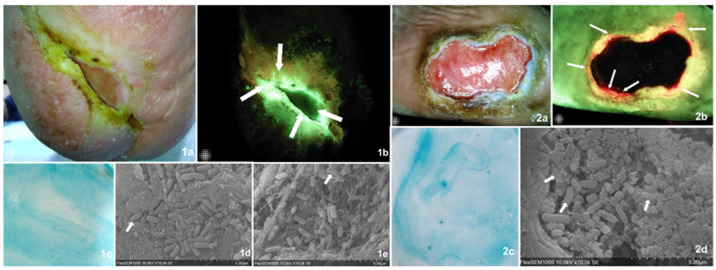
Visually describes, through two sample cases, the diagnostic tests undertaken on all samples including the validation methods and those under analysis. Images of findings from two case examples from the present study. Case 1. Diabetic foot ulcer present for over 12 months in a 63-year-old male patient. The wound area was 1.47 cm^2^ (MolecuLight^®^ digital measurement). (**1a**) Standard image of the wound which, according to the examiner, displayed clinical signs of biofilm including a slimy and shiny film on the wound surface that was easily detached and showed a favorable response to multimodal strategies of treatment. (**1b**) Fluorescence imaging identified areas of cyan bacterial fluorescence, which indicate the presence of *Pseudomonas aeruginosa* at concentrations at or above 10^4^ CFU/gr of tissue (arrows). (**1c**) The biofilm blotting did not produce a biofilm staining pattern, which meant this was a negative result. (**1d**,**1e**) The SEM readers agreed that matrix-associated bacterial clusters were present, suggestive of a biofilm. Arrows indicate areas of close matrix and bacteria colocalization, implying bacterial-derived matrix versus host-derived matrix. The microbiology results determined the presence of high bacterial loads including Pseudomonas aeruginosa and Corynebacterium striatum. This is an example of a true positive for both CBS and FL imaging and a false negative as found by blotting. Case 2. Diabetic foot ulcer present for over 12 months in a 49-year-old female. The wound area was 15.90 cm^2^. This subject was considered negative for biofilm presence based on clinical assessment, though the wound was also described as recalcitrant and tender to touch. (**2a**) Standard image of the wound. (**2b**) Fluorescence imaging identified areas of red bacterial fluorescence (arrows) particularly on the wound’s edge, indicating the presence of gram+/− bacterial concentrations at or above 10 ^4^ CFU/gr of tissue. (**2c**) Biofilm blotting did not produce the proper biofilm staining pattern. (**2c**,**2d**) The SEM readers agreed that matrix-associated bacterial clusters were present, suggestive of a biofilm. Arrows indicate areas of close matrix and bacteria colocalization, implying a bacterial-derived matrix versus host-derived matrix. The microbiology results determined the presence of medium bacterial loads including *Oligella urethralis*, *Pseudoglutamicibacter albus*, *Porphyromonas somerae*, *Brevibacterium ravenspurgense*, amd Corynebacterium striatum, amongst others. In this case, only FL imaging produced a true positive result, while both clinical assessment and biofilm blotting did not detect a biofilm.

Three easily implementable bedside biofilm detection methods were compared against the current validation SEM detection and microbiology. Two sample cases describing all assessment methods and their findings are found in Figure 2.

#### 2.3.1. Clinical Biofilm Assessment

Standard clinical assessment and digital wound measurement (MolecuLight**DX**, MolecuLight Inc., Toronto, ON, Canada) were conducted following the removal of dressings and gentle cleansing with normal saline-soaked gauze. Clinicians assessed the presence or absence of biofilm and reported one of two outcomes: either positive or negative for Clinical Signs of Biofilm (CSB) [CSB (+)/CSB (−)]. A suggested list of the specific clinical signs suggestive of biofilm most reported in the literature was provided based on the available relevant literature, including the wound biofilm identification diagnostic algorithm (BBWC) developed by Metcalf et al. [12,23,24].

The clinical signs of biofilm, as reported in the literature, are closely linked to its pathogenesis. Some authors theorize that biofilm exploits host inflammation to survive, inducing the upregulation of pro-inflammatory cytokines and causing persistent tissue destruction, thereby securing its own nutrient source at the host’s expense [25,26,27]. This contrasts with the inflammatory response in acute wounds, where inflammation occurs in typical stages and is primarily influenced by the host’s underlying conditions. Hurlow et al. highlighted some key differences in clinical signs in acute infections and chronic wounds, with many indicators in chronic wounds being usually associated with the presence of a biofilm. Some of these biofilm indicators include delayed wound healing, excessive moisture or exudate, wound breakdown or friability, unhealthy granulation tissue quality, excessive pain and/or malodor, discolored low-quality tissues, history of antibiotic treatment failure, culture-negative results despite signs of bacterial colonization or strong suspicion of local infection, and chronic wound non-resolution despite appropriate management of underlying comorbidities [12,23,24]. The BBWC algorithm [24] goes a step further and divides clinical signs of biofilm into visual and indirect indicators of biofilm. Visual indicators manifest once biofilm formation reaches a macroscopic stage, where the biofilm may appear as a shiny translucent substance on the wound surface or as an opaque loosely attached patch in certain areas. In some cases, it may have a viscous or gel-like consistency, and when certain pathogens like *Pseudomonas aeruginosa* or *Staphylococcus aureus* are present, the overlaying layer may exhibit a distinctive tinge, such as pale green or yellow. This superficial substance is difficult to remove through standard techniques like swabs, pads, or autolytic or enzymatic debridement, which dissolves key proteins that confer structural indemnity to the biofilm structure. It may even prevail following more aggressive removal methods such as sharp debridement [9]. Another visual cue noted is the reappearance of the substance or film shortly after removal, typically within a day or two. Indirect indicators include poor responsiveness to topical or systemic antibiotics, limited efficacy of antiseptic agents, and improved response to more aggressive physical interventions like sharp debridement. These criteria, along with the specific signs of biofilm noted above, collectively informed the clinicians’ classification of CSB as negative or positive. This decision was ultimately made by each individual clinician examining the wound, reflecting the reality of clinical practice.

#### 2.3.2. Biofilm Blotting Assay

A non-invasive technique was employed to identify the presence and spatial location of biofilm in a wound (Saraya^®^, Osaka, Japan) [28,29,30]. The wound-blotting technique involves applying a piece of nitrocellulose membrane to wounds for 10 s and then staining it with Alcian Blue dye. This process allows the visualization of biofilm on the wound surface, highlighting areas that still harbor biofilms.

In brief, a nitrocellulose membrane is applied to the wound, allowing it to absorb certain components of the biofilm’s EPS. The membrane is treated through a series of dyes and treatment solutions to visualize the presence of the biofilm’s EPS. As per the manufacturer’s instructions for use, a dark blue stain on the membrane after treatment indicates the presence of a biofilm. Due to supply constraints, only 35/40 patients received biofilm blotting. Therefore, accuracy measures were calculated for only 35 subjects for this diagnostic method against the validation methods.

#### 2.3.3. Fluorescence Imaging of Bacterial Loads

The fluorescence imaging device (MolecuLight**DX**, MolecuLight^®^, Toronto, ON, Canada) detects bacterial presence in wounds at potentially pathogenic concentrations based on fluorescence imaging principles [31,32,33]. In a darkened environment, standard and fluorescence images are captured at 8–12 cm from the wound. To capture a fluorescence image, the device emits a safe 405 nm violet light that excites endogenous bacterial components within the wound tissue. In response to this excitation wavelength, most bacterial species will emit a red fluorescent signal from the endogenous porphyrins that are produced in the heme pathway [34], while *Pseudomonas aeruginosa* will additionally fluoresce a distinctive cyan hue [32]. Cyan is best described as a bright white signal with a blue/green halo delineating its periphery. The fluorescence imaging device detects both red and cyan fluorescence signals when their bacterial burden reaches or surpasses loads of 10^4^ colony-forming units (CFU) per gram of tissue.

### 2.4. Validation Diagnostic Tests

Two of the most reliable and acknowledged methods of bacterial biofilm detection were employed in conjunction to determine the validation presence of biofilm in the study wounds. To classify a sample as biofilm-positive, the results from both methods needed to corroborate each other.

#### 2.4.1. Specimen Collection for Microbiology via qPCR Plus NGS and SEM Imaging

Specimen collection was performed via a sterile scalpel blade. Samples were 0.4 mm × 0.4 mm on average. Sample collection was performed as deep as possible to obtain a representative sample, but care was taken to avoid blood contamination. The specimen collection was performed after clinical assessment, blotting, and fluorescence imaging, but before debridement by an external technician (not involved with the interpretation process). This technician was blind to whether the sample was positive or negative in terms of fluorescence, blotting, and/or CSB.

The sample site location was chosen following the protocol outlined in Figure 1 (section IV). In short, on CSB-positive wounds where positive fluorescence signals were also found, the specimen was collected from an area of overlapping positive fluorescence and positive clinical signs. In the absence of fluorescence positivity, the selection of the sample site involved being based solely on clinical signs. On those wounds classified as CSB negative, the center of the wound was to be chosen unless a positive fluorescence signal was evidenced, in which case the specimen was collected from that area. Each collected sample was bifurcated, and one section was sent for microbiological analysis while the remaining section was sent for SEM imaging. The SEM sample was placed into a vial of SEM fixative (2.0% paraformaldehyde and 2.5% glutaraldehyde in 0.05 M sodium cacodylate buffer, pH 7.4) and labeled appropriately for blinded SEM assessment.

#### 2.4.2. Scanning Electron Microscopy (SEM) and SEM Image Interpretation

Sample processing was performed at the Nanoscale Biomedical Imaging Facility (NBIF, The Hospital for Sick Children, Toronto, ON, Canada). Briefly, the sample was fixed, dehydrated, mounted, and sputter-coated with 20 nm of gold palladium. Imaging was performed at Collaborative Advanced Microscopy Laboratories of Dentistry (CAMiLoD, Faculty of Dentistry, University of Toronto, Canada) on a high-resolution XL30 SEM (Hitachi High-Tech Corporation^®^ FlexSEM 1000 Scanning Electron Microscope, Tokio, Japan). The SEM technician was blinded to the bacterial load presence in the samples. Images were captured in a systematic and unbiased manner. A random starting location was chosen at magnification 50–60× and images at 2 k and 10 k magnification were captured at 12, 3, 6, and 9 o’clock. Each sample was analyzed for 30 min at maximum.

SEM image interpretation was performed by two independent readers (BRM and ACM) from different laboratories, also blinded to all other data points related to the specimen. The presence of bacterial clusters with the matrix located in close proximity to bacteria was interpreted as biofilm based on their expertise. True co-localization of the matrix and bacteria is most likely indicative of a bacterial-derived matrix, as opposed to a host-derived matrix, which is present regardless of infection or biofilm status. This observation generally held irrespective of the total bacterial load. Even when a relatively small number of cells were observed, the co-localization with the matrix increased the likelihood of identifying a biofilm.

#### 2.4.3. Quantitative Microbiology Using qPCR and Bacterial Speciation via Next Generation Sequencing (NGS)

The microbiology results provided a semi-quantitative measure of total bacterial load (TBL) based on 16S qPCR results. NGS provided bacterial speciation of the sample. TBL ≥ 10^5^ CFU/g was interpreted as possibly related to biofilm, while <10^5^ CFU/g was interpreted as not likely related to biofilm. This is the threshold used by the processing lab. The portion of the sample that was used for this purpose was deposited into an Eppendorf tube for transport to the processing lab. Transport was conducted immediately followed by processing of the sample first thing the next day.

### 2.5. Statistical Analysis

Exploratory endpoint analysis was performed to determine the accuracy, sensitivity, specificity, positive predictive value (PPV), and negative predictive value (NPV) of the various bedside biofilm detection methods.

McNemar tests (DTComPair R package) were employed to compare the sensitivity and specificity of the three methods in detecting biofilm. The comparison of predictive values (PPV and NPV) was performed by comparing relative predictive values as described in Moskowitz and Pepe (2006). To estimate the diagnostic accuracy characteristics, sample proportions and 95% confidence intervals (CIs) were used. All analyses were performed using R version 4.3.2.

## 3. Results

### 3.1. Patient Population

Overall, 40 of 45 eligible patients were included in the analysis herein, of which 80% were male. Their average age was 61.3 years (SD 13.9, [30–85]). Most wounds were diabetic foot ulcers (DFUs), accounting for 34/40 of the studied wounds. The remaining 8 were a combination of lower limb venous, arterial, neuropathic ulcers, and pressure injuries. The average wound area per digital measurement was 3.46 cm^2^ (SD: 2.54 cm^2^ [0.84–10.1]). All wounds were present for at least 4 weeks prior to enrollment. Wound duration was reported in 4-week increments (3 months, 3–6 months, 6–12 months, and >1 year), with most wounds lasting over 6 months. Table 1 details the study population’s demographics and wound characteristics.

### 3.2. Prevalence of Biofilm in the Study’s Population

Standard semi-quantitative microbiological analysis determined the presence or absence of bacterial loads above 10^5^ CFU/g. Evidence of the presence of a structural biofilm was determined by analysis of SEM images performed by two independent expert readers.

Bacteria clusters were categorized as matrix-associated (i.e., likely biofilm) if they were observed adjacent to matrix strands on the SEM images. Out of 40 collected samples, 82.5% (33/40) contained bacterial loads at or above 10^5^ CFU/g by microbiological analysis and 72.5% (29/40) were classified as containing matrix-associated bacteria based on SEM. Per our study, wounds were classified as biofilm-positive per validation assessment only when they exhibited both criteria for the primary analysis. Specifically, samples must have demonstrated clear evidence of matrix-associated bacteria through SEM analysis and possess a bacterial concentration exceeding 10^5^ CFU/g from quantitative microbiological analysis. Based on these criteria, a total of 62.5% (25/40) of the study wounds were identified as positive for biofilm. Among the remaining 15 samples, eight exhibited a bacterial load ≥10^5^ CFU/g as determined by qPCR + NGS yet there was no SEM evidence of bacterial presence in close proximity to the matrix. Consequently, these samples were classified as containing bacteria in a planktonic state or deemed “negative for biofilm” for accuracy measurement purposes. Two samples showed a total bacterial load below 10^5^ CFU/g based on microbiology, with no observable bacterial matrix presence in SEM images, thus constituting negative or control samples.

Additionally, four samples displayed evidence of bacterial matrix presence in SEM images but had a total bacterial load below 10^5^ CFU/g of tissue. These samples were labeled as “likely early immature biofilm” and were treated as “negative for biofilm” for accuracy measurement calculations in the primary analysis, but “positive for biofilm” when calculating against SEM findings alone. A secondary analysis and recalculation of the accuracy measures including sensitivity, specificity, PPV, and NPV was undertaken, in which samples with evidence of biofilm matrix under SEM were considered positive for biofilm; regardless of the bacterial load, this increased the number of positives to 29/40. This new calculation accounts for the fact that SEM remains a highly validated standalone tool for detecting biofilm and extracellular polymeric substances (EPS). However, diagnosing biofilm is not solely based on bacterial numbers; for example, immature biofilm may have a lower total bacterial load count well below 10^5^ CFU/g [35,36].

### 3.3. Diagnostic Accuracy of CSB, Biofilm Blotting, and Fluorescence Imaging

Three methods of bedside biofilm detection (i.e., Clinical Signs of Biofilm (CSB), bacterial fluorescence imaging (MolecuLight^®^), and biofilm blotting assay (Saraya^®^)) were tested for accuracy and reliability against microbiology and SEM as the gold truth. According to the BBWC clinical identification criteria, half of the patients recruited were positive for CSB (20/40), by design, in the hopes of recruiting both positive and negative biofilm samples. CSB and fluorescence imaging were assessed on all (40) wounds, while biofilm blotting was performed in 35/40 due to product supply constraints.

For the primary analysis, which was based on the gold truth criteria for biofilm presence including SEM positive and total bacterial loads ≥ 10^5^ CFU/g, the sensitivity of fluorescence imaging was significantly superior in biofilm detection at 84% (95% CI [64%, 95%]), versus just 45% (95% CI [28%, 64%]) for clinical assessment using CSB and 24% (95% CI [10%, 44%]) for blotting. The accuracy was also superior for fluorescence imaging (63%, 95% CI [46%, 77%]) versus 43%, 95% CI [27%, 59%] for CSB and just 29%, 95% CI [15%, 46%] for blotting assay. In contrast, fluorescence imaging did produce the lowest specificity of only 27% (95% CI [8%, 55%]), which was attributed to its ability to also detect planktonic bacteria. The specificity of CSB and biofilm blotting was 40% (95% CI [16%,68%]) and 64% (95% CI [35%, 87%]), respectively. All results are reported in Table 2.

Fluorescence imaging produced the highest number of true positives, identifying 21/25 biofilm samples (SEM+ and TBL ≥ 10^5^ CFU/gr of tissue) versus CSB (11/25) and biofilm blotting (5/21). PPV and NPV were comparable between the tests and not statistically significantly different; however, the NPV of fluorescence imaging was higher over the two other methods (50% versus 30% for CSB and 36% for blotting). This suggests fluorescence imaging may be more reliable when attempting to rule out the presence of bacteria, including biofilm.

A sub-analysis examined the association between wound duration and the presence of biofilms, as determined by the criteria used in this study (positive SEM and ≥10^5^ CFU/g microbiology). Statistical analysis was limited due to the sample size. But, in short, there was no consistent trend or relation between the number of wounds found to be positive for biofilm per the validation methods and the wound duration that could signal a higher presence of biofilm as the duration of the wound increased. In fact, there was no difference in the distribution of wounds positive or negative for biofilm of different durations (*p* = 0.39). The bedside detection methods assessed herein yielded similar results, with no trend in the distribution of positive/negative wounds in relation to wound duration. However, a greater number of false negative and positive results were evidenced in wounds of over 12 months duration. For blotting, this meant a greater number of false negatives, and for fluorescence imaging, a greater number of false positives.

A secondary analysis using SEM as the gold standard for biofilm detection yielded slightly different results. The sensitivity of fluorescence imaging decreased from 84% to 79%, while CBS and the blotting assay remained consistent but low (45% and 25%, respectively). The specificity for fluorescence imaging also dropped from 27% to 18%, while the other methods remained stable. The positive predictive value (PPV) increased for all methods, while the negative predictive value (NPV) decreased for all. Interestingly, diagnostic accuracy remained stable across all three methods of biofilm detection. Table 3 provides a detailed summary of these results. Fluorescence imaging continued to reveal the highest number of true positives, identifying 23/29 biofilm samples (SEM+ irrespective of TBL) versus CSB (13/29) and biofilm blotting (6/24). False negatives, which can lead to missed opportunities to treat, were predominant in the blotting (18/24) and CBS (16/29) methods but constituted a small portion of fluorescence imaging results (6/29).

Lastly, considering the potential relevance of bacterial presence, especially at significant concentrations, due to their potential to form or indicate the presence of biofilm and their role in chronic inflammation and healing impairment, we determined the diagnostic accuracy of each biofilm detection method based solely on microbiology results [6,23,37,38]. In this case, wounds that harbored bacterial burden over 10^5^ CFU/g were considered positive irrespective of the SEM findings. In this analysis, the accuracy of fluorescence imaging increased to 78% (95% CI [62%, 89%]), while the accuracy of CSB (43%, 95% CI [27%, 59%]) and biofilm blotting (29%, 95% CI [15%, 46%]) remained the same or decreased. The sensitivity of all detection methods remained virtually unchanged. The sensitivity of fluorescence imaging (85%, 95% CI [68%, 95%]) remained statistically significantly higher than CSB (45%, 95% CI [28%, 64%], *p* < 0.001) and biofilm blotting (24%, 95% CI [10%, 44%], *p* < 0.001). Specificity, however, did increase in the case of fluorescence imaging, i.e., to 43% (95% CI [10%, 82%]), but decreased significantly for CSB, i.e., to 29% (95% CI [4%, 71%]) and slightly for biofilm blotting, i.e., to 50% (95% CI [12%, 88%]), *p* > 0.05) (Table 4).

## 4. Discussion

The present study serves as a bridge between basic science exploration of biofilm and its clinically relevant and actionable bedside detection. We examined the prevalence and detection of biofilm in chronic hard-to-heal wounds using established validation methods and compared the accuracy of three practical bedside biofilm detection techniques. Biofilm was present in 63% of the study wounds per the gold standard method of SEM identification consistent with positive semi-quantitative microbiology. The presence of bacterial matrix per SEM alone can be an indicator of biofilm, in which case the prevalence of biofilm would have increased to 73%. In any case, we found a considerable prevalence of biofilms in the chronic wounds studied herein. Prior research links biofilm to the interruption of the healing cascade [12,39,40] and ultimately to the onset of complications. If bacterial burden and biofilm are not promptly addressed, overt or covert localized infection can quickly mount and rapidly progress into more serious issues such as osteomyelitis, gangrene, sepsis, amputation, and death [41,42].

The presence of bacteria, particularly in biofilm form, can induce hyperinflammation in wounds leading to the production of toxins and destructive enzymes that damage tissues [19,20,37]. Biofilm communities possess more sophisticated protective and adaptive mechanisms compared to planktonic bacteria. They tolerate antibiotic treatments and other forms of intervention such as wound debridement and cleansing more robustly than free-floating bacteria, making them particularly challenging. Some researchers propose that the mere presence of a biofilm community not only increases the risk of hyperinflammation and wound recalcitrance but also requires the presence of an upregulated pathogenic bacterial species to trigger such detrimental effects [23,43,44]. Notably, the inflammatory state of a wound is determined by different factors in acute versus chronic wounds. In acute wounds, the inflammatory response is more influenced by host factors and tends to be more exuberant, whereas in chronic wounds biofilm elicits a lower-grade and persistent inflammatory response [23]. Intrinsic bacterial conditions and characteristics such as microbial load, the bacterial species present, their synergistic interactions, and their mutual potentiation contribute to the origin, growth, perpetuation, and virulence of a biofilm [23,45,46,47,48]. Additionally, underlying comorbid conditions and environmental factors further damage tissues, impair healing, and contribute to bacterial proliferation and the further spread and intensification of their effect on healing and tissue degradation [38,39,41,42].

Removing bacteria from wounds seems to directly re-establish the healing process; however, the diagnostic hurdles associated with early bacterial detection, particularly biofilms, make delivering timely and effective wound care challenging. The most broadly used means to detect inflammation and infection at the bedside is clinical assessment for signs and symptoms; however, this method is far from ideal due to its low sensitivity and specificity [31]. In the case of bedside biofilm detection, as implemented in the present study, the clinical signs and symptoms are highly subjective (e.g., a slimy shiny translucent layer on the wound surface that is easily removable but quickly reappears) [23]. Other clinical cues are unspecific and unable to definitively confirm biofilm presence (e.g., failure to heal despite standard care with local infection management for 45 days, continual necrotic tissue formation and friable granulation tissue in the wound bed, ineffective antimicrobial treatments, and partial wound healing followed by recurrence or breakdown) [24,40,44]. Laboratory-based methods of biofilm detection, such as light microscopy (LM), confocal laser scanning microscopy (CLSM), atomic force microscopy (AFM), or scanning electron microscopy (SEM), offer more objective diagnostic advantages. These provide high-resolution images of biofilm structure and morphology and insights into microbial spatial distribution, rather than relying heavily on subjective assumptions. However, these techniques demand specialized equipment, require the use of dyes, fluorophores, and invasive probes, and involve time-consuming sample preparation and analytical expertise, altogether rendering them impractical for many settings [49]. Similarly, molecular techniques like Polymerase Chain Reaction (PCR), quantitative PCR (qPCR), and metagenomic sequencing offer DNA-based detection of biofilm-forming microorganisms, yet demand specialized equipment, reagents, and bioinformatics expertise, thus limiting their accessibility to research or specialized clinical laboratories. Moreover, specificity is still lacking [49].

The important qualities of the methods assessed in the present study include real-time easily implementable bedside biofilm detection. This supports timely interventions by providing immediate actionable information that translates into intentional treatment strategies [31,50], outcomes improvements, and reductions in complications [51,52]. We found that, of the three methods assessed, bacterial fluorescence imaging exhibited the highest sensitivity in biofilm detection (84%), albeit with the lowest specificity (27%), likely due to its inability to differentiate between planktonic bacteria and biofilm. Biofilm blotting demonstrated the highest specificity (64%), but with the lowest sensitivity (24%), and clinical assessment using CSB fell within the mid to low range for both sensitivity and specificity. Physician time commitment and the workflow impact of biofilm detection varied by method. Clinical assessment is part of routine wound care, meaning CSB does not require additional clinician time. Fluorescence imaging requires the time to position the patient and properly adjust the lighting. The fluorescence information appears in real-time, and images are captured and must be interpreted by the trained clinician. Biofilm blotting is added to the time required to blot the wound, to develop the membrane with the various dyes and washes, and to interpret the results. On average, this process took slightly longer for us than the reported 2 min in the literature [53]. CSB, fluorescence imaging, and biofilm blotting all were performed at the bedside during the visit, emphasizing their practicality compared to the gold-standard laboratory-based method of SEM with microbiology confirmation. Both SEM results and microbiology results are only available days after the patient visit. SEM analysis was very involved and required specific niche expertise and protocol optimization. Sample preparation was completed alone over 2 days, as is standard [54], and SEM imaging was performed in batches and capped at 30 min per sample in this study. Experts were also required to examine and interpret the SEM images, independent of the imaging. The time and complexity of SEM imaging are well reported and are a barrier to use [54]. The average time to receive the microbiology results was 7 days (ranging from 4–10 days).

These findings, however, do not unequivocally identify a superior method across all scenarios. It is crucial to also highlight what represents actionable information and holds the utmost relevance in the clinical context of chronic wound management. Expert consensus indicates that the localization of biofilm within the wound and its thorough and repetitive debridement yields the best results [2,40]. These ideas also apply to bacteria in planktonic form. So, while identifying biofilm specifically may hold scientific interest, which calls for a highly specific method, clinically, both biofilm and planktonic bacteria are important and should be addressed similarly [37,38,40]. Both can cause harm and delay healing unless they are identified, disturbed, and mechanically removed [37,38,40]. In that case, a highly sensitive tool may present as more useful compared to a highly specific tool as it is less likely to miss any form of bacteria. Bedside tests used at the outset of treatment planning should prioritize high sensitivity over high specificity because the primary goal is to minimize the number of missed true positives, thus decreasing the chance of missing those wounds in need of intervention. This objective is achieved effectively by fluorescence imaging in the present study; while the blotting method offers sufficient specificity, it may serve as a secondary confirmatory step in the diagnostic process. This is particularly relevant in the clinical context where bacteria in both planktonic and biofilm forms can cause diverse (i.e., chronic and acute) infections and wound healing impairment [23]. This has been objectively described by recent research that indicates the presence of elevated protease activity, which is linked to bacterial pathogenesis and inflammation, in relation to all bacterial forms [55].

Traditional models for biofilm formation theorize that microbes follow a linear and incremental trajectory, first attaching to surfaces and then producing adhesins, transitioning from reversible to irreversible attachment. Intracellular c-di-GMP levels determine the planktonic or biofilm state, microcolonies begin to form through EPS formation, and then later, mature biofilms disperse cells due to factors like nutrient deficiency or mechanical stress [56]. As per this framework, as bacteria become incrementally established, the number of detectable microbes increases, and clinical expression becomes more apparent. However, this five-step model is now being challenged by recent studies, which suggest limitations in its applicability. For instance, Sauer et al. [36] propose an alternative model where bacteria can switch forms between planktonic and biofilm based on an interplay between environmental factors and intrinsic bacterial regulation. In this scenario, bacterial communities are versatile and do not follow a linear trajectory of accumulative communal formation. This model suggests that it is important to detect any bacteria present, regardless of the state in which it is found. It is important to note that while bacteria in a planktonic state pose a lesser threat compared to an established biofilm community, they should not be underestimated. Costerton et al. [6] emphasized that bacteria grow differently in the lab than in their natural habitat. In in vivo conditions, unlike in in vitro conditions, bacteria are more likely to form biofilms due to environmental threats such as nutrient limitations. Therefore, any bacterial form found in significant concentrations could be considered a potential biofilm in formation.

The need for effective bedside and easily implementable biofilm detection methods in wound care cannot be overstated. These methods would assist clinicians in promptly identifying biofilm presence during routine wound assessments and this timely detection is paramount for initiating targeted interventions aimed at disrupting biofilm formation. This personalized approach is instrumental in optimizing treatment outcomes, improving healing, and reducing the risk of complications and recurrence. Of the three methods assessed herein, clinical assessment holds some value in aiding the detection of biofilm, but due to the low sensitivity of CSB, clinical opinion should be supplemented by more objective and sensitive techniques. Fluorescence imaging employs a handheld non-contact device to emit specific wavelengths of light, revealing endogenous bacterial fluorescence patterns associated with bacteria and biofilms [32,33,34,57,58]. This non-invasive method provides actionable information by pinpointing areas affected by biofilm and/or high bacterial loads, aiding targeted treatment. Wound blotting captures biofilm-related biomarkers from wound exudate, offering insights into biofilm presence albeit with limitations in sensitivity and specificity that may lead to potential false negatives. While there is no available substitute for highly specialized SEM, implementing bedside bacteria and biofilm detection methods could enhance resource utilization and healthcare efficiency. In that sense, the high NPV of fluorescence imaging enhances confidence in ruling out biofilm presence, while still displaying a good sensitivity for biofilm detection against this validation method. This, added to its identification of other bacterial forms, has great practical utility in guiding treatment decisions and potentially reducing unnecessary interventions while improving outcomes, as has been demonstrated by other studies [51,52,59].

## 5. Study Limitations

- The biofilm prevalence reported here is likely an underestimation due to a small sample size;
- The microbiological analyses herein were semiquantitative, whereas quantitative analysis could have provided more insights, particularly in the cases that were inconclusive for biofilm;
- The threshold used in the microbiological analysis was set as 10^5^ CFU/g of tissue due to processing lab standards. If this threshold had been set lower, the results of this analysis could provide more information. The MolecuLight device detects bacteria at a lower threshold (starting at 10^4^ CFU/g), which differs from this threshold. However, it is worth noting that a number of publications suggest that lots of 10^5^ CFU/g and above have clinical significance [60,61];
- Due to product supply constraints, only 35/40 subjects included were exposed to wound blotting. This meant that sensitivity and specificity were calculated with a slightly smaller sample size. When sample sizes are small, the confidence interval around the sensitivity and specificity widens, indicating greater uncertainty. To what extent this affected the outcomes in this study is uncertain;
- While efforts were made to choose the most suitable validation method, there is currently no consensus on the definitive method for detecting biofilms. If an alternative diagnostic standard emerges in the future, it could impact the accuracy of the current bedside measures that have been selected.

## 6. Conclusions

In exploring the diagnostic performance of three methods for detecting wound biofilms at the bedside, we observed a high biofilm prevalence consistent with other reports [62,63], which highlights the importance of its detection in clinical practice. The evaluation of three methods of bedside biofilm detection indicates that, given current models of biofilm formation where bacteria fluidly switch between planktonic and biofilm states, a highly sensitive method capable of detecting bacteria in various states such as fluorescence imaging has an advantage over CSB and biofilm blotting. Not discounting the confirmatory qualities of other more specific methods such as blotting, fluorescence imaging seems to be the tool that derives more actionable information from an early stage. Timely clinical management of biofilm hinges on its early and accurate detection—a challenge with laborious benchtop detection methods such as PCR and SEM. Overall, we found fluorescence imaging to be the most accurate and, importantly, the most clinically applicable bedside biofilm detection method. In general, practical time-effective methods that offer actionable information that enhance outcomes and support proactive action should be favored.

## Figures and Tables

**Table 1:** Demographics and wound characteristics of the study’s population.

Wound Characteristic	Value
Number of wounds (n)	40
Age	
Mean (SD)	61.3 (13.9)
[range]	[30–85]
Sex (n) [%]	
Male	32 [80]
Female	8 [20]
Fitzpatrick Score (n) [%]	
I–II	25 [62.5]
III–IV	13 [32.5]
V–VI	2 [5]
Wound type (n) [%]	
DFU	34 [85]
VLU	3 [7.5]
ALU	1 [2.5]
PU	1 [2.5]
Neuropathic Ulcer	1 [2.5]
Wound Location (n) [%]	
Forefoot	15 [37.5]
Midfoot	11 [27.5]
Heel	7 [17.5]
Ankle	7 [17.5]
Wound duration (n) [%]	
1–3 months	8 [20]
3–6 months	9 [22.5]
6–12 months	9 [22.5]
12+ months	14 [35]
Wound area (cm^2^)	
Mean (SD) [range]	3.46 (2.54) [0.84–10.08]

**Table 2:** Diagnostic test performance against the combined results from two gold-truth methods (SEM and microbiology).

Parameters	Value, % [95 CI%]		
	FL	CSB	Blotting Assay
Accuracy % [range]	63 [46, 77]	43 [27, 59]	40 [24, 58]
Sensitivity % [range]	84 [64, 95]	44 [24, 65]	24 [8, 47]
Specificity % [range]	27 [8, 55]	40 [16, 68]	64 [35, 87]
PPV % [range]	66 [47, 81]	55 [32, 77]	50 [19, 81]
NPV % [range]	50 [16, 84]	30 [12, 54]	36 [18, 57]
DOR% [range]	1.87 [0.36, 9.88]	0.54 [0.14, 1.98]	0.57 [0.12, 2.67]

**Table 3:** Diagnostic test performance against SEM results alone.

Parameters	Value, % [95 CI %]		
	FL	CSB	Blotting Assay
Accuracy % [range]	63 [59, 89]	43 [41, 44]	37 [21, 53]
Sensitivity % [range]	79 [60, 92]	45 [26, 64]	25 [10, 47]
Specificity % [range]	18 [2, 52]	36 [11, 69]	64 [31, 89]
PPV % [range]	72 [53, 86]	65 [41, 85]	60 [25, 88]
NPV % [range]	25 [3, 65]	20 [6, 44]	28 [12, 49]
DOR% [range]	0.88 [0.1, 4.95]	0.48 [0.1, 2]	0.59 [0.12, 3.03]

**Table 4:** Diagnostic test performance against microbiology results alone.

Parameters	Value, % [95 CI %]		
	FL	CSB	Blotting Assay
Accuracy % [range]	78 [62, 89]	43 [27, 59]	29 [15, 46]
Sensitivity % [range]	85 [68, 95]	45 [28, 64]	24 [10, 44]
Specificity % [range]	43 [10, 82]	29 [4, 71]	50 [12, 88]
PPV % [range]	88 [71, 96]	75 [51, 91]	50 [12, 88]
NPV % [range]	38 [9, 76]	10 [1, 32]	12 [3, 31]

## Data Availability

Data may be available upon request.
